# Risky Drinking Cultures Among Affluent Youth in Sweden

**DOI:** 10.3389/fpubh.2022.867802

**Published:** 2022-07-08

**Authors:** Linda Hiltunen, Pia Kvillemo, Youstina Demetry, Johanna Gripenberg, Tobias H. Elgán, Charlotte Skoglund

**Affiliations:** ^1^Department of Social Studies, Linnaeus University, Växjö, Sweden; ^2^Department of Clinical Neuroscience, Stockholm Prevents Alcohol and Drug Problems, Center for Psychiatry Research, Karolinska Institutet and Stockholm Health Care Services, Stockholm, Sweden; ^3^Department of Clinical Neuroscience, Center for Psychiatry Research, Karolinska Institutet and Stockholm Health Care Services, Stockholm, Sweden; ^4^Department of Pharmaceutical Biosciences, Uppsala University, Uppsala, Sweden

**Keywords:** adolescents, affluence, alcohol, alcohol problems, social identity

## Abstract

There is a growing scientific interest in drinking behavior among young people in affluent areas, who report higher levels of alcohol consumption compared to youth in less privileged areas. This phenomenon has been observed in several Western countries. The research has been dominated by variable-oriented analyses and has presented interesting explanations, but there has been little research into these young people's own experiences of and attitudes toward alcohol consumption. To develop interventions targeting this group, we need to understand their lifeworld. This study aims to develop an in-depth understanding of the high alcohol consumption among young people in affluent areas and how they themselves experience it. In the spring of 2019, we conducted 20 in-depth interviews with adolescents in upper secondary school (aged 15–19) in one of the most affluent area in Sweden. The empirical material was analyzed thematically. Theoretically, the phenomenon is understood by relating to social identity processes and considering the group's material, social and cultural means through Bourdieu's metaphors of capital. We found that affluent youth link their social identities to alcohol consumption. Alcohol is a social beverage that opens social networks and contributes to a sense of community. The consumption of alcohol gives experience capital leading to status in this context, with clear norms and expectations governing alcohol consumption. Parties are arranged in protected spaces where young people are free to drink out of the adults' sight. Affluent youths also have considerable purchasing power which contributes to drinking, and they are socialized into a pre-existing adult alcohol culture characterized by a liberal view on alcohol. Finally, when alcohol consumption escalates, the youths perceive that it is difficult to get adequate help from the adult world. The findings are important for future preventive interventions for subgroups of adolescents at high risk for heavy drinking.

## Introduction

Over the last two decades, there has been a growing scientific interest in the drinking behavior of young people in areas of affluence in several western countries (1–6). Adolescents who grow up in affluent areas have in fact been identified as a new group at risk for heavy drinking (7, 8). The phenomenon has also been observed in a Swedish context where the present study takes place (9, 10). Alcohol is associated with a wide variety of problems, harms and disorders (11–14). It is therefore important to deepen our knowledge of drinking patterns among affluent youth, who report the highest levels of alcohol consumption.

Research that has examined alcohol consumption among youth in affluent areas mainly builds on survey studies and a range of interesting results have been presented. However, very little qualitative research has been conducted into the attitudes toward and experiences of alcohol consumption in this group of high consumers of alcohol. Previous studies have highlighted that this knowledge gap can be due to difficulties in gaining access to the social elite in affluent areas, which have been described as “gated communities” (15, 16). Qualitative data that offers in-depth knowledge about the lived experiences of alcohol consumption in this group is clearly needed.

In the present study, adolescents are interviewed in an affluent municipality in Sweden where, according to previous studies, alcohol consumption is comparatively high (9). The aim of this study is to develop an in-depth understanding of high alcohol consumption as it is experienced by young people in an affluent area in Sweden. The results constitute an important basis for future development of targeted intervention programmes to reduce alcohol consumption in this new group at risk for heavy drinking.

### Alcohol Consumption Among Young People in Affluent Areas

In Sweden, the proportion of young people who consume alcohol has decreased since the early 2,000 s (17). Moreover, the amount of alcohol has also decreased among those who drink (18). In parallel with this positive development, alcohol consumption continues to be high among adolescents who grow up in affluent areas in Sweden (9). The present study is situated in one of Sweden's socioeconomically strongest municipalities in the capital region of Stockholm, where previous surveys have shown that 80 per cent of the girls in their second year of upper secondary school and 68 per cent of the boys in the same year have been drunk, which is 10–15 per cent higher than for Stockholm as a whole. The proportion of youths who report that they are high consumers of alcohol is also higher in this municipality, with 37 per cent of the girls (compared to 27 per cent for Stockholm as a whole) and 42 per cent of boys (compared to 29 per cent) reporting this (19).

### Earlier Explanations for Young People's Heavy Drinking

Previous studies have offered several explanations for why affluent youth drink more than others. Firstly, it is well-known that alcohol consumption increases when youth have their own money to spend (3, 20). Purchasing power also seems to be particularly important among youth in the Nordic countries where the cost of alcohol is high (21).

Secondly, previous research has highlighted a much higher prevalence of alcohol use in wealthier areas among adult populations compared to areas with lower incomes (22, 23). A possible explanation is thus that affluent youth are socialized into the adults' alcohol consumption patterns (24). Studies show, for example, that adolescents in affluent areas have greater access to alcohol and that the norms among parents are more permissive and liberal (2, 3). That children are offered alcoholic beverages or take them for their own use without permission, when there is plenty of alcohol in the home (25), has also been suggested to be a plausible mechanism (3). Even though there is a higher prevalence of alcohol use in wealthier areas, it is important to emphasize that alcohol problems are more common among both youths and adults in more disadvantaged areas (4, 26, 27). Affluent youth's frequent consumption of alcohol and greater risk of alcohol intoxication but low level of alcohol problems is a paradox (3, 28). One possible explanation is that youths in affluent neighborhoods use alcohol in a manner that is compatible with a rather health-oriented lifestyle and that they are also able to handle complex expectations fairly well (21). These possible explanations have, however, not been established empirically and more research that can shed light on the mechanisms behind the high level of alcohol consumption is needed.

Lastly, affluent youth may be at risk of maladaptive outcomes and adjustment problems due to both their own and their parents' high expectations (29–31). Therefore, they are at risk of experiencing distress resulting in substance use as a way of “self-medicating” to cope with achievement pressure and isolation from parents (8). The disconnection from parents—both literal and emotional—has been highlighted as resulting from the demanding careers of adults in affluent areas (8).

Studies that have observed high levels of alcohol consumption among affluent youth mainly build on surveys that cannot shed light on the mechanisms and local norms that maintain the high level of alcohol consumption on the individual and group level (3, 21, 29). The present study thus makes an important contribution.

### Social Identities and Resources in Affluent Areas

In adolescence, drinking is a normative practice and an important component in identity formation for many youths (32, 33). Against this background, social identity serves as an analytic tool in this study, seeking to shed light on the characteristics that individuals share with others in a group (34). Social identities are the definitions, attributes and social positions that an individual is ascribed in interaction with others (35). How we see ourselves and how others see us are thus important elements in the process of social identity formation. The formation of social identities is also an ongoing process (36), not least in one's younger years when belonging to different groups can be subject to negotiation. Social identities are also constructed by differentiating between groups (36). To construct an “us,” we also need a “them” (35). Social identities are thus hierarchical as some group identities appear to be better than others, depending on the context and situation.

Research that has exclusively focused on young people's identity formation has been criticized for not considering the individual's material, social and cultural means to freely choose their lifestyle and who they want to be (37). Young people can rarely freely choose to construct whichever social identities they want; rather, there are not only limitations to what is possible but also local rules for what is desirable in a specific context. To shed light on the individual's material, social and cultural means of identity construction in relation to alcohol consumption, we use Pierre Bourdieu's metaphors of capital. Bourdieu proposes different forms of capital that can be exchanged for recognition and respect in a given context (38). The present study considers economic capital (material assets), social capital (networks, group membership and friendships) and cultural capital (education, language, style and habits) (38). Bourdieu's metaphors of capital have mostly been used as analytical tools to study social reproduction. In this study we use the metaphors of capital to understand how alcohol cultures are reproduced in an area that is economically, culturally and socially elevated. The term alcohol culture, which is central in this study, is used to describe young people's collective attitudes, learned perceptions and behaviors connected to alcohol (39).

## Materials and Methods

### Approach

The study's point of departure has been to focus on young people's perspectives and experiences. The qualitative method was deemed to be the most appropriate to capture the complexities and nuances in young people's attitudes to alcohol in a specific context. Through in-depth interviews, we could deepen our understanding of the youths' own attitudes and experiences of alcohol consumption.

### Study Setting

The study is set in Sweden, which in comparison to many other countries is characterized by strict regulations of alcohol and illicit drugs (40, 41). Alcoholic beverages (>3.5% alcohol content by volume) can only be bought at the Swedish Alcohol Retailing Monopoly “Systembolaget” by people 20 years of age or older, or at licensed premises (e.g., bars, restaurants, clubs) from the minimum age of 18 years. All use of illicit drugs is criminalized. In Sweden, active alcohol prevention work is carried out and young people are an important target group for various preventive measures (32). Political action plans and preventive programmes have been formulated at the political level (42) and Swedish schools are commissioned to convey knowledge about the health risks of alcohol (40).

The study was carried out in one of the most affluent municipalities in Sweden (here referred to as “Villaholm”), focusing on young people from families in the highest socioeconomic strata. The annual family income is 45 per cent above the national mean and the municipality has the highest educational level of all Swedish municipalities, with 58 percent of the population (25 years and over) having graduated from university and holding professional degrees, as compared to the national average of 26 per cent (43). That the municipality has plenty of resources can also be illustrated by the fact that its upper secondary schools have high admission points and produce high grades. In this municipality, 83 per cent complete their upper secondary education with a degree within 3 years compared to 66 per cent in the country as a whole. In addition, 3 years after graduating from upper secondary school, 65 per cent (compared to 41 per cent nation-wide) have commenced higher education (44).

### Recruitment

In the spring of 2019, the principals of all three upper secondary schools in the municipality were contacted and gave their consent that we could recruit youths for the study through the schools.

Information and an invitation to participate in the study was published on the schools' online platforms. Students communicated their initial interest in participating to the assistant principal. Upon consent, the assistant principal forwarded the mobile phone numbers of eligible students to the research team. That way, the research team came into contact with 20 upper secondary school students who expressed their interest in participating in the study. Everyone who expressed their interest in participating in the study was included.

Of the 20 interviewees, 11 were males and 9 females, and they were between 15 and 19 years old (mean age: 17). All of them were enrolled in university preparatory school programmes (13 in social science and 7 in natural science programmes). The interviewees had different attitudes toward alcohol. Nine can be categorized as heavy drinkers in the sense that they drink every weekend or more frequently. Eight described a more moderate attitude by drinking alcohol only every other weekend; three of them used to drink a lot of alcohol but had at the time of the interview taken on a more restrictive attitude. A smaller group (3 participants) described that they actively reject alcohol. Capturing different perspectives and perceptions of alcohol was desirable because it can nuance our understanding of the phenomenon under study.

### Data Collection

The interviews took place in the autumn of 2019. We discovered that the participants' time was limited and decided—in accordance with the interviewees' wishes—to conduct phone interviews, which lasted ~60 min on average. The preferred telephone interviews strengthened the anonymity of the participants, and our assessment is that it provided openness in addressing sensitive topics, such as speaking about drinking habits. The in-depth interviews aimed to deepen our understanding of the meaning and significance the youths attach to alcohol and alcohol consumption. The semi-structured interviews addressed four main themes: (1) The alcohol culture in Villaholm; (2) One's own relation to alcohol; (3) One's friends' and family's relation to alcohol; and (4) What support young people need to reduce alcohol consumption. All interviews were recorded and transcribed verbatim.

### Data Analysis

A thematic analysis was used to identify, analyse and report patterns (themes) within the data based on the premise that the researchers approach the data set with a specific question in mind (45). The process of analysis commenced with reading the whole interview transcripts several times to familiarize ourselves with the data corpus. In line with the procedures of thematic analysis, the guiding principle in our first readings was to identify and highlight the parts of the material that touched upon and offered answers to our research question (45), i.e., why alcohol consumption is high in this particular context. Note was made of all material in the data that in some way was concerned with this theme.

Once we had discerned the data that was to be used for this analysis, the next step was to thoroughly and carefully code each fragment of the empirical material with the aim of summarizing and organizing the data into meaningful groups for further analysis (45). These codes were then sorted into potential themes, which were continuously refined and further specified in the reading and processing of the material. The codes and themes were identified in an inductive or “bottom-up” process (45), which means that the themes emerge from the data rather than from the theoretical interest (46). The analysis finally resulted in six separate themes that are presented in the results section.

### Ethical Considerations

The study was approved by the Swedish Ethical Review Authority (dnr. 2019-02646). All procedures concerning confidentiality, data management and informed consent were followed. To protect the participants' privacy, all names used in this article are fictitious. When quotes are presented in the results section, the purpose is to illustrate the phenomenon being described and to show how interpretations are made and conclusions are drawn. The quotes that are presented have been chosen because they most clearly shed light on the youths' way of reasoning. All quotes have been translated from Swedish and some have been edited slightly to avoid irrelevant words and too colloquial language.

## Results

Six themes emerged as important components of the high level of alcohol consumption among affluent youth: (1) Community and belonging; (2) The alcohol norms of parties; (3) Protected spaces; (4) Resources for consumption; (5) A liberal adult alcohol culture; and (6) Level of parental involvement.

### Community and Belonging

All of the interviewees describe alcohol consumption as highly normalized in Villaholm. “In upper secondary school almost everyone drinks,” Rebecca states and goes on to convey that it “is more the rule than the exception to drink.” Most were introduced to alcohol already in their final years of compulsory school, and in upper secondary school drinking is well-developed. Alcohol consumption in upper secondary school is also described as more sophisticated than the “reckless” drinking of the earlier years. Drinking alcohol can be interpreted as experience capital that young people are expected to have acquired once they reach upper secondary school. “I guess it's part of the culture to party every weekend,” Julia says and maintains that “in Villaholm, alcohol is a way in to (join) social circles.” Alcohol is described as an integral part of youth culture and there are clear expectations of drinking together. Drinking is also perceived to be a key component of at all being allowed in and taking part of social life in Villaholm:

If you want to have a social life in Villaholm, it is incredibly difficult if you don't drink at parties. […] I personally would say that it is very difficult to have a social life without alcohol here. […] I think you feel socially excluded if you don't drink […] that's why I feel like it's very important [to drink]. Partying, it's a kind of culture [in Villaholm] and to be part of this social life is extremely important for us youth.—Alice.

Alice puts into words a shared perception that social life includes alcohol and that it is difficult to stand outside of this normative practice without sanctions. Alcohol consumption is consistently described as a way of socializing, creating a sense of community and not least establishing new social networks, which Carolina sheds light on:

There were a lot of parties, especially at the start of the first year, considering that… Well but there were so many new people and everyone wanted to party and welcome each other.—Carolina.

As described above, the youths often describe that the parties, including alcohol, become a way of finding one's bearings in the new life in upper secondary school. Drinking becomes a way of getting to know one another, letting go of one's self-control and getting together without really knowing each other that well-beforehand. In that way, alcohol functions as an invitation rite and a social lubricant when young people become acquainted with one another. Alcohol becomes a sign of community in a new life and a way of constructing social identities in interaction with others. Eric sheds light on this by using himself as an example:

You're quite insecure and you don't know who you are. Alcohol then often becomes a way of finding somewhere to fit in. […] If you drink then you kind of have a guaranteed place at the table.—Eric.

The quote illustrates that Eric is insecure and looking for a clearer social identity. He tries to strengthen his own identity claim by drinking alcohol together with others, which also can be seen in the other youths' descriptions. The quote also illustrates how drinking can be exchanged for recognition and that alcohol becomes a symbol of belonging to a group. Youths who let themselves go and party away also get a larger social network, which in turn strengthens their position among their peers. This interpretation finds support in Isabell's reasoning: “I have friends at several schools, so I go to all of the schools' parties.” Her words witness that Isabell has a large social network that gives her access to the spaces where parties are arranged. One conclusion is that young people who via their social capital manage to make many contacts with their peers also get more opportunities to party.

### The Alcohol Norms of Parties

In Villaholm, alcohol consumption is strongly associated with glamorous parties that need to be arranged by a host and come with clear expectations of both the host and the guests. Norms guide what is appropriate and inappropriate. For example, an invitation is required and one is expected to contribute to the party with alcoholic beverages:

The parties that are the most fun are often the ones where everyone brings their own alcohol. […] Because the host of course thinks it's more fun if everyone is hammered and you have a fun evening. […] When you drink alcohol, you become more self-confident, can let loose and laugh at anything and the party becomes more fun […] you're expected to, like, be a fun guest.—Philip.

In this quote, Philip sheds light onto a tacit agreement between the host and the guests to bring their own alcohol and to contribute to the festive mood. There is an expectation to be relaxed and have fun together, which alcohol contributes to. Showing up empty-handed or declining alcohol are violations of the norm. The participants agree that the purpose of the alcohol is to let go of inhibitions, to open up and elevate the mood: “With alcohol in your body, it's not as stiff […] you dance around and do crazy things,” Simon explains for instance. The social interaction flows more smoothly and several participants emphasize explicitly that they want exactly these “effects” of alcohol. With a drink in hand they can also identify with one another and legitimize their own participation in the party. It is important to fully participate and be part of the party, and not stand on the sidelines: “It's not that much fun to see people walk around and have fun, and then you just stand there on your own and kind of just look around,” Ludvig explains. The study's participants see a clear value in getting drunk together.

Apart from tacit agreements to drink, the participants also describe a more explicit peer pressure to get drunk, which everyone must deal with. Those who get drunk go unnoticed at the party while those who do not drink risk being “detected” and subjected to pressure from their peers:

People are on to you, like, [they say] “come on now, take a little.” Yeah, people say: “Damn you're boring!” […] It's quite a lot of pressure, you're supposed to drink. You just do it.—Agnes.

Agnes describes that those who do not drink are encouraged to do so in the party setting. All of the participants find that this collective correction and social pressure is difficult to counter. As described by Alice in an earlier quote, those who go against the drinking culture also risk having their social identity questioned, which further drives up alcohol consumption. The participants also emphasize that there is a significant risk of being entirely excluded from social events in Villaholm if you do not drink:

You don't want them [those who don't drink] at a party where you're the host. […] You're of the opinion that you can't have fun without alcohol, so you don't invite non-drinkers. […] Those who don't drink […] can go and create their own capital in the same way as we who drink do, can't they?—Elvira.

In the quote, Elvira highlights that the norms and values are narrow and intolerant. One interpretation is that alcohol consumption is not only socially desirable, but also an obligation for those who want to be part of the collective community. In the empirical material, there are three individuals who have rejected drinking and who confirm that they are not invited to the parties:

It is much more difficult to be invited to a party when people know that you don't drink, because then people think that you're quite boring as a person.—Lina.
I've never been to a party. On Mondays after the weekend, I just hear that a lot of weird, stupid things have happened. […] Yeah, but it's a little odd. You feel a little excluded […] so it's like a social thing that you miss out on.—Alexander.

These quotes illustrate that those who do not confirm to the alcohol norm risk being sanctioned. The quote presented by Lina highlights a shared perception that the non-drinkers are considered “boring,” which is a consistent pattern within the interviews. One interpretation is that the non-drinkers are attributed a lower cultural capital among the young people in Villaholm because they adhere to the expected normative behaviors. Consequently, they become somewhat lonely and rather isolated, as described by Alexander. Against this background, one interpretation is that alcohol serves as a sorting principle for inclusion and exclusion in Villaholm.

Yet another norm to be dealt with at parties is the amount of alcohol to be consumed. Some, but not all, describe that the goal of drinking is to become obviously drunk, as explained here:

You have worked hard in school and then you just have to really let loose and drink. […] Almost all of my friends have basically been, like, unconscious. […] Many have fainted, slept outdoors during the winter […]. Several have had their stomachs pumped. Ludvig.

Ludvig here describes binge drinking, which is associated with known health risks. Getting into a drunken stupor in the way that Ludvig describes, however, does not apply to all—it mostly concerns boys who have adopted a clear alcohol identity. One interpretation is that it is about pushing the boundaries of what the body can handle. Girls also get drunk, but it happens in a more controlled manner. In the quote, Ludvig highlights yet another norm that is well-worth addressing. He, just like other youths, finds that alcohol consumption is a way of relaxing after a pressured school day, which presents a normative practice. The interviews convey that education is highly valued and that achievements and high grades are considered important. Against this background, one interpretation is that alcohol and intoxication form a contrast and a welcome change from everyday life. In this way, alcohol also serves as a temporary reward and an escape from everyday obligations.

One conclusion is that the influence of peers and the alcohol norms that young people create together form a social system that drives alcohol consumption. Experiences of alcohol represent a cultural capital where a clear alcohol identity contributes to gaining respect and recognition from one's surroundings, while those who do not adopt the given alcohol norms risk being collectively excluded. Against this background, it is difficult for the individual to say no to alcohol.

### Protected Spaces

The results show that there is a culture of gathering around parties in Villaholm. Apart from the school parties, which are arranged by the student union at each school, the youths also talk about private parties. From the interviews it emerges that many families have economic capital (several properties and country houses) which, in turn, gives the youths spatial capital (empty apartments and houses) to arrange private parties: “During the summer, the parents are in the countryside, so then you have the houses to yourself,” Simon explains. The homes are protected spaces where the youths can be left alone and can select who they choose to include in their social group. Isabell has made the following observation:

There are certain people whose parents almost never are home and they have big houses. They often have parties 4–6 times a year and let more than 100 people come to the parties. And then their parents aren't home at all and don't notice. Isabell.

The quote highlights that a handful of absent parents are enough for parties to be arranged for a relatively large number of individuals. To ensure order at these large events, which up to 60–80 people attend, sometimes older youths are needed to keep watch. There is a clear pattern in the interviews that the arrangement of a private party is associated with status and recognition.

Another pattern is that the youths in this sample suggest that parents in Villaholm have a demanding working life that is characterized by long working days and travel. This means that parties also can be arranged when parents are away on work trips:

Many people I know have very loving parents… […] but maybe they are not there all that much. […] It's not that the parents don't care, but it can be more like they maybe travel a lot and work.—Alice.
In Villaholm, parents are away more often […] which makes it more accessible for parties […] and that you have the money for it.—William.

The spatial capital and the absence of parents who are busy with their working lives is seen as problematic by many of the youths, although it is emphasized throughout that the parents are loving and that they care a lot about their children. One interpretation is thus that young people whose parents are busy with their own professional careers have larger spatial and financial means to take on the role of hosting a party.

### Resources for Consumption

None of the participants who drink alcohol mention finances as an obstacle for consumption. Rather, the expectation that one can procure alcohol on one's own is clearly expressed:

As you get older, alcohol becomes more available […] Then you're expected to be able to get it yourself.—Philip.

Since young people in upper secondary school cannot legally buy alcohol, it is mainly purchased from illegal distributors of alcohol on social media who deliver directly to them or to the party:

When you come to a party, people will come up to you and ask: “Do you want to buy some alcohol? We have a dealer who is on the way in a van right now.”—Ludvig.

The availability of alcohol is not perceived to be a problem and the norm is to show high purchasing power. The availability of alcohol gives further incentive to bring your own alcohol to the party or to buy some when the opportunity arises.

### A Liberal Adult Alcohol Culture

The young people in this sample suggest that there is a distinct alcohol culture where the adults also gather around dinner invitations and parties, which include alcohol. Watching their parents drink alcohol in festive contexts is part of many of the participants' experience of growing up:

You see your parents drink alcohol and that is so normalized. If they have a longer dinner then they'll down a couple of bottles of wine. […] It's the whole culture, not least in Villaholm, that drinking is something nice. […] Then you have an inner will to grow up yourself and be older than you really are. So you imitate what you see the older ones do. Alcohol is like a clear symbol that you've become older.—Eric.

The youths describe alcohol as symbolizing becoming an adult and taking on a clearer adult identity. One interpretation is that the youths' drinking habits partly can be explained by them being socialized into an already existing alcohol culture shouldered by adults. Some of the participants problematised the parents' liberal attitude toward alcohol as being negative, even though they frequently drank themselves:

I think that the parents […] in Villaholm are starting to ease up a little when it comes to views on alcohol. Because there are quite a lot who… they care of course about their son or daughter drinking […] but it's not the world's biggest deal. They kind of accept it and I can think that's negative.—William.

William describes parents' liberal attitude toward alcohol as negative. The young people in this study express that they wished for a more restrictive parental attitude and suggest that when the parents' restrictions are eased, this can contribute to an increased alcohol consumption, as explained here:

**Elvira:** The first time they noticed I was drunk […] they were angry and that's justified.
**Researcher:** Did it lead to any consequences?
**Elvira:** No, my parents don't believe in consequences. […] I was told off and we had a dialogue on why I should not [drink].
**Researcher:** How did you react?
**Elvira:** Yeah, I thought it was just so weird. I just… “but I'll stay home if you want me to,” like that. I still think it's very weird.

As described above, the youths express a desire for parents to take on a more restrictive attitude and thereby help them limit their alcohol consumption. What emerges from the interviews is that the youths interpret this accepting attitude as meaning that they are free to drink and that their parents even are expecting them to drink alcohol: “Well, it's almost expected of you to try it and become drunk some time,” Fanny states for example. One interpretation and conclusion is that young people are influenced by and are socialized into the alcohol norms represented by parents and other adults in Villaholm.

### Level of Parental Involvement

In Villaholm, many young people who drink do not face any direct resistance from their parents, which means that the parents' role in intervening in alcohol consumption is limited. The participants report that the parents in many cases probably know that their children drink, but that they do not understand the extent of it. Agnes explains: “If my parents saw me on a night out they would think that I drank too much.” The pattern is that adolescents try to hide that they become obviously drunk at parties, which is expressed by Fredrik: “You wouldn't be happy to come home too drunk in front of your parents.” One interpretation is that young people are open about drinking, but that they hide heavy drinking from their parents. In addition, youth in this study also highlights a norm system where the heavy drinking must be kept as a secret from parents. The norm system is maintained by the fact that any interactions with somebody's parents about drinking imply a loss of status. Therefore, they do not ask their parents for help, for example if somebody becomes obviously drunk:

You're just so afraid to lose your status. […] That's why you don't want to be the one who calls her or his parents. Then you've ruined your whole school experience, so that's not an option.—Eric.

Calling somebody's parents is a violation of norms that can have consequences for one's social identity and can lead to one's position in the community of peers being questioned. At the same time, the youths express that they at times need the help of adults, for example when friends have developed risky patterns of use or become too drunk. But they have nowhere to turn, as expressed by Fanny: “It would be easier to help your friends if you knew what to do. You don't want to tell on them.” Many also perceive that even when they know about hazardous use, parents do not act and take hold of the problem:

Parents don't dare to deal with it, because they don't want it to be about their own children. I know a parent who found drugs in their child's room but didn't want to mess things up for them. They make threats but nothing happens. Parents are very naive.—Isabell.

The participants report that both adolescents and parents in Villaholm are anxious about possible alcohol or drug problems becoming known by their surroundings or being registered in the health care system. Contact with the authorities is to be avoided and they hold the view that alcohol problems preferably are to be dealt with within the closed sphere of the family:

People are afraid of going to seek health care because they don't want it [alcohol and drug use] in their medical records. There can be problems with that… […] if it's written down somewhere, everything can be ruined.—Sophia.

One interpretation is that there is an obvious risk that the support that authorities can offer young people is not as accessible for children who grow up in privileged areas. The participants express that they are worried that alcohol problems (or drug use) that is documented in health care or the judiciary system could present an obstacle for studies abroad and future professional careers. Opportunities are perceived to be greater with a “clean” record. As one of the participants describes:

There were four guys who were caught smoking weed. They were, like, the popular guys. It's just gone downhill for them ever since. […] They can't get a job when they graduate. They can't study abroad any more until the demerit goes away. […] One of them was going to get a really good job, but it was retracted in connection with the demerit. So their opportunities disappeared.—Emma.

This demonstrates that any status and popularity that has been worked up is destroyed by police intervention, with the consequence that one's social identity is questioned and becomes less legitimate. One interpretation is that contact with the authorities—not least the judiciary system—can be especially hard on young people in privileged areas because their future career plans risk being lost. Being publicly exposed entails a risk of being cut off both from one's social identity and from promised economic, cultural and social capital.

## Discussion

### Reflections on the Findings

The aim of this study was to develop an in-depth understanding of the norms, attitudes and experiences behind the high alcohol consumption among young people in affluent areas. The study takes an approach that considers the meaning of alcohol in relation to processes of identity formation. A theoretical contribution is made in that the study also considers the individual's material, social and cultural means of creating a successful social identity in line with Bourdieu's metaphors of capital (38). While there is much literature on alcohol consumption among adolescents, empirical research into the experiences of this specific population of young people is limited even though affluent youth has been observed to be a group that is at risk for heavy drinking (7, 8).

The study shows that affluent youth link their social identities to alcohol consumption. We found six themes that emerged as important components of the high level of alcohol consumption among affluent youth. *Community and belonging* highlights that alcohol is a social beverage that opens networks and contributes to a sense of belonging. *The alcohol norms of parties* illustrate that there are clear norms and expectations governing alcohol consumption when parties are arranged. Parties are also arranged in *protected spaces* where young people are free to drink out of the adults' sight. Affluent youths also have considerable *resources for consumption* which contributes to drinking. Also, they are socialized into a pre-existing *liberal adult alcohol culture* characterized by a liberal view on alcohol. Finally, when alcohol consumption escalates, the youths perceive that it is difficult to get adequate help from the adult world and what is needed is *more parent and adult intervention*.

Our findings contribute to the literature in several ways: by giving insight into how an alcohol culture can manifest itself among affluent youth; by showing that local norms can play a role in alcohol consumption; by shedding light onto the mechanisms that maintain previously identified relationships; and by nuancing the findings of previous research on alcohol consumption among affluent youth.

### Identity Construction in Relation to Bourdieu's Social, Cultural and Economic Capital

In line with earlier studies, this study shows that alcohol consumption is a normative practice in adolescent years and an important component in identity construction (32, 33). One conclusion is that there is a pronounced alcohol culture in Villaholm that permeates social life. In upper secondary school, drinking is the norm and most have acquired this experience capital. The youths associate alcohol with parties, glamor, relaxation and becoming an adult. The study shows that drinking is a way of socializing and alcohol consumption is described as an entry ticket to groups of friends and a larger social circle. In line with Bourdieu's framework the analysis shows that by establishing relationships—via alcohol—one's social capital increases, which in turn can be exchanged for larger networks, social belonging and popularity. Previous studies show that this, in turn, increases the odds of being invited to more parties and drinking more alcohol (16, 47). Alcohol consumption thus promotes young people's identity construction and social capital.

In line with earlier studies, the influence of peers and the system of norms that young people themselves create in this specific context constitute important parts in explaining the high level of alcohol consumption (32, 47, 48). The findings show that there are clear local drinking norms in Villaholm. Party hosts expect their guests to bring their own alcohol, get drunk and contribute to the party spirit. By adopting the alcohol norm and developing a clear alcohol identity, you gain respect in your surroundings and avoid having your social identity questioned. Individuals with a clear alcohol identity do not problematise these expectations but comply with the norms that surround party life. When considering the ability to take on the norms of party life and its rituals as a form of cultural capital, in Bourdieu's meaning, conformity becomes easier to understand. Our contribution to research lies in shedding light onto experiences of alcohol as cultural capital, with a clear system of sanctions that drive alcohol consumption.

Our findings also qualify the common assumption that economic factors have a crucial role in high alcohol consumption (3, 21). The youths in our study are economically privileged and have their own money to spend, which earlier studies have linked to high alcohol consumption (20).

This also corroborates the concept of income elasticity, where a higher income is related to increased consumption (49, 50). Moreover, Bourdieu's concept of economic capital reveals that the youth can buy as much alcohol for their own use as they like, at the same time as drinking gives experience capital leading to status in this local context. Through liberal alcohol consumption, a social identity without financial limits can be staged, which contributes to the risk of heavy drinking among youths in affluent areas.

In addition, affluent youths have access to spatial capital where parties can be arranged in protected spaces without risking that alcohol consumption is detected. Private parties without adult supervision and control are associated with high status, recognition and successful identity construction, which further may contribute to an increased alcohol consumption in Villaholm.

### Socialization Into Adults' Alcohol Consumption Patterns

Previous research has highlighted that the demanding environment in affluent areas can cause ill health among young people, which is associated with substance use (8, 29, 31). Villaholm is described as a demanding environment where youths are high achievers and value education. Alcohol consumption as a way of dealing with perceived ill health is, however, not particularly salient in this study. Rather, alcohol is used as a contrast to relax from the duties of everyday school life. Alcohol is considered a culturally legitimate means of escape, making it fully acceptable and rational to relax with the help of alcohol even though adolescents (under the age of 18) still are under the legal drinking age.

Our findings confirm earlier assumptions that affluent youths are socialized into adults' drinking cultures (21, 24). The participants describe a pronounced alcohol culture among adults which adolescents imitate when they stage adult identities. Children and adolescents are social beings and they are influenced by the culture they grow up in. This socialization mainly occurs by observing the adults' way of drinking and seeing that parents have a permissive attitude toward alcohol (3, 25). The agreement that it is acceptable to drink a little at parties instead results in adolescents drinking a lot has also been confirmed by earlier studies (2, 51). Youths who come home drunk are not punished in any clear way, which is perceived that drinking is something acceptable. One conclusion is that the lack of intervention—in the sense of parental resistance—contributes to young people's alcohol consumption. The study's results cannot, however, confirm earlier hypotheses that socialization takes place by affluent youth being offered alcohol in their own homes or that the parents would purchase alcohol for them [cf. (25)]. In this regard, the parents rather take on a strict attitude.

Our findings also show that it is difficult to ask parents or adults for help when it comes to alcohol. If there are any alcohol or drug problems to be dealt with, this happens primarily within the family and without any contact with the authorities, which is typical of families in socially and economically elevated areas (15, 30). Previous studies have shown that affluent families may be particularly concerned about privacy (16). A paradoxical conclusion, in line with earlier studies, is that children from the wealthiest families have less access to the services that society can offer young people with risky alcohol use (16).

### Future Interventions

The study's findings are important for future preventive interventions for this subgroup of youth at high risk for heavy drinking. Existing methods do not suit this group and they are not inclined to ask the authorities for help (16, 30). One important finding is that the youths urge their parents to revisit their laissez-faire attitude toward adolescent alcohol use (2). The study's findings shows that parents have influence on the drinking behavior of their adolescent children. It also highlights the importance of parental monitoring and support to protect adolescent from alcohol use, which has been confirmed in earlier studies (52). Findings from this study also highlight that the influence of peers is very important for alcohol consumption. This suggests that it is important to problematise the alcohol norm rather than individual drinking habits when future preventive programmes are developed. An important question to take into consideration in such intervention work is how we can get adolescents to lower their alcohol consumption when it is so strongly tied to the process of social identity formation. One possible way is to develop interventions where alcohol consumption can be discussed from a norm-critical perspective in dialogue with adolescents, rather than getting each individual to drink less. In preventive work, it is also important to consider the context in which drinking occurs since norms and ideas are likely to differ between different countries and between different cities and neighborhoods (21, 48). To achieve its desired effects, it is important that alcohol prevention work is something that young people can relate to (53).

### Reflections on Limitations and Strengths

Qualitative interviews are generally conducted in person. In this study, they were conducted using mobile phones which could be considered as a limitation. Telephone based interviews may be inferior to face-to-face interaction since they do not allow for interpretation of more discrete signals such as body language and indirect communication. In addition, technical problems may interfere and disrupt the interviews (54). However, it is important that the participants are given influence in how and where the interview takes place, whereupon the wishes to conduct telephone interviews were accepted. The flexibility of using phones meant that the participants were free to choose place for the interview and did not have to physically meet the interviewers. Using telephone interviews had also other advantages. As previous research has shown (54) our assessment is that the anonymity of the participant was strengthened, and the power relations were balanced, which in turn contributed to the interviewees generously sharing their experiences about alcohol. Therefore, our perception is that the advantages of this approach outweigh the possible limitations.

Since the study is comparatively small, it makes no claim for the results to be generalisable to a larger population. The study was also carried out in a specific privileged context, which means that the results cannot necessarily be translated to other populations and the external validity is low. A strength of the study is that it offered a unique opportunity to explore the drinking culture in a privileged neighborhood and that the participants generously shared their experiences. To our knowledge, few have previously gained access to socially and economically elevated areas to carry out studies of this kind. Another strength of the study lies in that we have examined both the meaning alcohol has for young people and the risks it entails for them. For example, we can conclude that alcohol is a beverage that is associated with health risks, but also a symbol for the communication of identities, and that adolescents shape their alcohol identities in relation to what is possible when it comes to financial, social and cultural resources. A final limitation is that adolescents with more severe health or psychosocial problems may have refrained from participating, biasing the results toward more psychosocially stable adolescents.

### Continued Research

The present study confirms that adolescents in privileged areas present a risk group for harmful alcohol use. An important element of future research would be longitudinal studies that follow up on the negative effects of alcohol among youth in affluent areas. Studies that shed light on alcohol cultures in several more privileged areas are also needed since norms probably develop in different ways in different countries, cities and local contexts (21). Another aspect which is not addressed in depth in this study is the use of drugs, which half of the participants have experience of. The normalization of party drugs is a concerning result not least because drug liberals seem to have the finances for consumption and perceive of the drugs as a status symbol, which requires more attention in continued research.

## Data Availability Statement

Collected data will be available from the Centre for Psychiatry Research, a collaboration between the Karolinska Institute and Region Stockholm. But restrictions apply to their availability, as they were used under ethical permission for the current study and so are not publicly available. However, data are available from the corresponding author upon reasonable request and with permission from the Centre for Psychiatry Research.

## Ethics Statement

Written informed consent was obtained from the minor(s) for the publication of any potentially identifiable images or data included in this article.

## Author Contributions

LH contributed to conceptualization, methodology, data curation, formal analysis, writing original draft, and review and editing. PK contributed to methodology, investigation (data collection), data curation, formal analysis, validation, review and editing, and funding acquisition. YD contributed to conceptualization, investigation (data collection), review and editing, and project administration. JG contributed to review and editing, and funding acquisition. TE contributed to review and editing. CS contributed to methodology, investigation (data collection), data curation, formal analysis, validation, review and editing, funding acquisition, and supervision. All authors approved the submitted manuscript version.

## Funding

The work was funded by the Alcohol Research Council of the Swedish Alcohol Retailing Monopoly (grant no. 2018–0010). The funding body had no role in study design, data collection, analysis, data interpretation or writing the manuscript.

## Conflict of Interest

The authors declare that the research was conducted in the absence of any commercial or financial relationships that could be construed as a potential conflict of interest.

## Publisher's Note

All claims expressed in this article are solely those of the authors and do not necessarily represent those of their affiliated organizations, or those of the publisher, the editors and the reviewers. Any product that may be evaluated in this article, or claim that may be made by its manufacturer, is not guaranteed or endorsed by the publisher.

## Supplementary Material

Appendix A An interview guide document, including themes and questions used when conducting the interviews.
